# The Copper Efflux Regulator CueR Is Subject to ATP-Dependent Proteolysis in *Escherichia coli*

**DOI:** 10.3389/fmolb.2017.00009

**Published:** 2017-02-28

**Authors:** Lisa-Marie Bittner, Alexander Kraus, Sina Schäkermann, Franz Narberhaus

**Affiliations:** Microbial Biology, Ruhr University BochumBochum, Germany

**Keywords:** AAA^+^ proteases, proteolysis, Lon, ClpXP, ClpAP, CueR, copper homoeostasis, MerR family

## Abstract

The trace element copper serves as cofactor for many enzymes but is toxic at elevated concentrations. In bacteria, the intracellular copper level is maintained by copper efflux systems including the Cue system controlled by the transcription factor CueR. CueR, a member of the MerR family, forms homodimers, and binds monovalent copper ions with high affinity. It activates transcription of the copper tolerance genes *copA* and *cueO* via a conserved DNA-distortion mechanism. The mechanism how CueR-induced transcription is turned off is not fully understood. Here, we report that *Escherichia coli* CueR is prone to proteolysis by the AAA^+^ proteases Lon, ClpXP, and ClpAP. Using a set of CueR variants, we show that CueR degradation is not altered by mutations affecting copper binding, dimerization or DNA binding of CueR, but requires an accessible C terminus. Except for a twofold stabilization shortly after a copper pulse, proteolysis of CueR is largely copper-independent. Our results suggest that ATP-dependent proteolysis contributes to copper homeostasis in *E. coli* by turnover of CueR, probably to allow steady monitoring of changes of the intracellular copper level and shut-off of CueR-dependent transcription.

## Introduction

Copper is a trace element required as cofactor for full functionality of several enzymes, such as cytochrome c oxidase of the respiratory chain (van der Oost et al., 1994). The intracellular copper concentration must be strictly maintained since elevated copper levels are toxic for the cell, e.g., by generation of reactive oxygen species (Rensing and Grass, 2003; Grass et al., 2011). In *Escherichia coli* two copper efflux systems, the Cue and the Cus system, adjust the intracellular copper level to the cellular demand (Rensing and Grass, 2003; Rademacher and Masepohl, 2012). While the Cus system operates under anaerobic conditions, the Cue system is predominantly active under aerobic conditions (Outten et al., 2001). CueR, the key regulator of the Cue system, activates transcription of the copper tolerance genes *copA* and *cueO* (Outten et al., 2000; Stoyanov et al., 2001). CopA is a P-type ATPase located in the cytoplasmic membrane and pumps monovalent copper ions (Cu^+^) into the periplasm (Petersen and Møller, 2000; Rensing et al., 2000). The multi-copper oxidase CueO is located in the periplasm and oxidizes Cu^+^ to the divalent form, Cu^2+^, which is not able to pass the inner membrane by simple diffusion (Grass and Rensing, 2001; Rensing and Grass, 2003).

The transcription factor CueR is a member of the MerR family named after the mercury resistance regulator MerR (Brown et al., 2003). Proteins of this family typically form homodimers and are comprised of three characteristic domains: the N-terminal DNA-binding domain, the central dimerization helix, and the C-terminal metal-binding domain (Brown et al., 2003; Changela et al., 2003). CueR contains two copper-binding cysteines in its metal-binding domain (C112, C120), which are essential for covalent binding of monovalent copper ions. An active CueR homodimer, binding two Cu^+^ ions (holo-CueR), induces the expression of *copA* and *cueO* by binding to their promoter regions which induces torsional transformations in the DNA conformation (Changela et al., 2003; Chen et al., 2003; Stoyanov and Brown, 2003; Philips et al., 2015). By kinks and undertwisting, the DNA switches from a B-form into an A-form-like conformation that allows access of the RNA polymerase. The metal-free CueR dimer (apo-CueR) is also able to bind to the promoter region resulting in a tight DNA conformation, which represses *copA* and *cueO* expression (Philips et al., 2015).

CueR binds copper with high affinity (Changela et al., 2003). An open question is how CueR-mediated expression of copper detoxification systems is turned off when necessary or how the cellular CueR pool is maintained to allow continuous sensing of the actual intracellular copper level. Several studies have implicated a role of proteolysis in the regulation of metal homeostasis (Lu and Solioz, 2001; Solioz, 2002; Lu et al., 2003; Solioz and Stoyanov, 2003; Liu et al., 2007; Pruteanu et al., 2007; Pruteanu and Baker, 2009). Regulated proteolysis is a universal post-translational strategy adapting the existing protein pool to the cellular demand. In *E. coli* five different ATP-dependent proteases (AAA^+^ proteases, ATPases associated with a variety of cellular activities), namely ClpXP, ClpAP, HslUV, Lon, and FtsH, are responsible for quality control of proteins as well as for the regulated turnover of intact proteins (Baker and Sauer, 2006; Sauer and Baker, 2011; Bittner et al., 2016). AAA^+^ proteases are comprised of two functional domains, the ATPase and protease domain. While the proteases ClpP and HslV associate with separate ATPases to form ClpXP, ClpAP, or HslUV complexes, the two domains of Lon and FtsH are encoded by a single gene. The ATPase domain is needed for ATP-dependent unfolding and translocation of a substrate into the proteolytic chamber of the protease domain, in which the substrate is degraded (Bittner et al., 2016; Sauer and Baker, 2011). AAA^+^ proteases recognize their substrates via exposed recognition motifs, so-called degrons and also adaptor proteins can be involved in recognition (Sauer et al., 2004; Baker and Sauer, 2006; Gur et al., 2011, 2013; Sauer and Baker, 2011). An example for proteolysis of proteins involved in metal homeostasis is the MerR-like regulator ZntR, which binds zinc (Changela et al., 2003) and activates expression of the zinc exporter ZntA (Brocklehurst et al., 1999; Outten et al., 1999). ZntR is a substrate of the Lon and ClpXP proteases in *E. coli* (Chivers, 2007; Pruteanu et al., 2007; Pruteanu and Baker, 2009). Moreover, the metallochaperone CopZ from *Enterococcus hirae* and the *Saccharomyces cerevisiae* proteins Ctr1p (plasma membrane transporter for high-affinity copper uptake) and Mac1 (copper-sensing transcriptional activator) are degraded upon increased copper levels (Ooi et al., 1996; Zhu et al., 1998; Lu and Solioz, 2001; Solioz, 2002; Lu et al., 2003; Solioz and Stoyanov, 2003; Liu et al., 2007). Here, we report proteolysis of the metalloregulator CueR by Lon and the ClpP machineries in *E. coli*.

## Materials and methods

### Bacterial strains and growth conditions

*E. coli* strains used in this study are listed in Table 1. Cells were grown in liquid LB, 2YT, or M9 minimal medium in a water bath shaker (180 rpm) or on LB agar plates at 30 or 37°C. When required, antibiotics were used as follows: ampicillin (Amp) 100 μg/ml, chloramphenicol (Cm) 25 μg/ml, kanamycin (Kan) 50 μg/ml, or tetracycline (Tet) 10 μg/ml.

**Table 1:** ***E. coli*** **strains used in this study**.

***E. coli*** **strain**	**Relevant characteristics**	**Source**
DH5α	*supE44*, Δ*lacU169* (Y80*lac*ZDM15), *hsdR17, recA1, gyrA96, thi1, relA1*	Sambrook and Russell, 2001
K12	wild type	Bachmann, 1972
MC4100 (RH166)	MC4100 Δ*ara*/Δ*leu, lac^−^*	Becker and Hengge-Aronis, 2001
Δ*lon*	RH166, *lon:Tn10*	Barembruch and Hengge, 2007
Δ*clpP*	MC4100 Δ*clpP::kan*	Schmidt et al., 2009
BW25113	F*^−^*, Δ(*araD*-*araB*)*567*, Δ*lacZ4787*(*::rrnB-3*), λ*^−^, rph-1*, Δ(*rhaD-rhaB*)*568, hsdR514* (CGSC # 7636)	Baba et al., 2006
Δ*clpA*	BW25113; F*^−^*, Δ(*araD-araB*)*567*, Δ*lacZ4787*(::*rrnB-3*), λ*^−^*, Δ*clpA783*::kan, *rph-1*, Δ(*rhaD-rhaB)568, hsdR514* (JW0866-1; CGSC # 8898)	Baba et al., 2006
Δ*clpX*	BW25113; F*^−^*, Δ(*araD-araB*)*567*, Δ*lacZ4787*(::rrnB-3), Δ*clpX*724::kan, λ*^−^, rph-1*, Δ(*rhaD-rhaB*)568, *hsdR*514 (JW0428-1; CGSC # 8591)	Baba et al., 2006
Δ*hslUV*	MC4100, *hslUV::kan*	Barembruch and Hengge, 2007
W3110	F^−^, IN(*rrnD-rrnE*)1	Tatsuta et al., 1998
Δ*ftsH*	W3110, *zad220::Tn10 sfhC21*Δ*ftsH3::kan*	Tatsuta et al., 1998
MG1655	F^−^, λ^−^, *rph-1*	Bachmann, 1972
KY2981	MG1655 Δ(*clpPX-lon*)*1196::cat*, Δ*hslVU1172::tet, sulA2981*	Kanemori et al., 1999
WOII260A	*lacI*^*q*^, *lacZ_*WJ*16_*, Δ*cueR*, Φ(*copA*-*lacZ*)	Outten et al., 2000
WOII248B	BW25113, Δ*cueR*	Outten et al., 2000
BL21[DE3]	F^−^, *ompT, gal* (*dcm*) (*lon*), *hsdSB* (*rB^−^mB^−^*), λ[DE3]	Studier et al., 1990
CH1019	X90*ssrA:cat*[DE3] Δ*yefM-yoeB::kan*	R.T. Sauer

### Construction of plasmids

Plasmids and oligonucleotides used in this study are listed in Tables 2, 3, respectively. Recombinant DNA techniques were performed using standard protocols (Sambrook and Russell, 2001). *E. coli* DH5α cells served as cloning host. For construction of inducible CueR expression plasmids, genomic *E. coli* K12 DNA was used as template for PCR amplification of the *cueR* gene for full-length or C-terminally truncated CueR variants. The PCR product was cloned into pASK-IBA5(+) or pASK-IBA3 via primer-derived restriction sites to create pBO2584, pBO2585, pBO2860, or pBO2862, respectively. CueR variants with amino acid substitutions were generated by QuikChange® PCR using pBO2584 as template and mutagenized primers to create pBO2591, pBO2595, or pBO4800, respectively. For construction of pBO3687, a plasmid encoding constitutively expressed CueR, the *cueR* gene was amplified from genomic *E. coli* K12 DNA and cloned into pACYC184 via primer-derived restriction sites. This plasmid was used for QuikChange® PCR to create constitutively expressed CueR_C112S variant (pBO4801). All cloning results were confirmed by sequencing.

**Table 2:** **Plasmids used in this study**.

**Plasmids**	**Relevant characteristics**	**Source**
pASK-IBA5(+)	Amp^r^, P/O_tet_, *tetR*, encodes for N-terminal Strep-tag fusions	IBA GmbH
pASK-IBA3	Amp^r^, P/O_tet_, *tetR*, encodes for C-terminal Strep-tag fusions	IBA GmbH
pACYC184	Low copy number cloning vector, Cm^r^, Tet^r^	New England Biolabs
pBO2584	pASK-IBA5(+) derivative encoding Strep_CueR (N-term. Strep-tag)	This study
pBO2585	pASK-IBA3 derivative encoding CueR_Strep (C-term. Strep-tag)	This study
pBO2591	pASK-IBA5(+) derivative encoding Strep_CueR_R18A_ (N-term. Strep-tag)	This study
pBO2595	pASK-IBA5(+) derivative encoding Strep_CueR_A78C_ (N-term. Strep-tag)	This study
pBO2860	pASK-IBA5(+) derivative encoding Strep_CueR_ΔC5_ (N-term. Strep-tag)	This study
pBO2862	pASK-IBA5(+) derivative encoding untagged CueR	This study
pBO3687	pACYC184 derivative encoding for constitutive expression of CueR	This study
pBO4800	pASK-IBA5(+) derivative encoding Strep_CueRC112S (N-term. Strep-tag)	This study
pBO4801	pACYC184 derivative encoding for constitutive expression of CueR_C112S	This study
pBO1115	pET19b derivative encoding His_6__CspD	Langklotz and Narberhaus, 2011
pET21b-Lon	Amp^r^; P_T7_; encodes for Lon with a C-terminal His_6_-tag fusion	R.T. Sauer

**Table 3:** **Oligonucleotides used in this study**.

**Name**	**Variant**	**Template**	**Sequence (5′-3′)**	**Plasmid**
Strep-CueR-IBA.fw	*cueR*	gDNA	AAAAGAATTCAAACATCAGCGATGTAGCAAAAATTACC	pBO2584
CueR.rv			TTTTAAGCTTTCACCCTGCCCGATGATGAC	
Cuer-Strep.fw	*cueR*	gDNA	AAAAGAATTCAACATCAGCGATGTAGCAAAAATTACC	pBO2585
CueR-Strep.rv			TTTTCCATGGGGCCCTGCCCGA	
CueR_R18A.fw	*cueR_R18A*	pBO2584	TGACCAGCAAAGCAATTGCCTTCTAT	pBO2591
CueR_R18A.rv			CTTCTCTTCATAGAAGGCAATTGCTTTGC	
CueR_A78C.fw	*cueR_A78C*	pBO2584	GGCACAGCTGCGATGTCAAACG	pBO2595
CueR_A78C.rv			GCCGTTTGACATCGCAGCTGTG	
CueR.fw	*cueR*_Δ*C5*	gDNA	AAAATGTACAAACATCAGCGATGTAGCAAAAATTACCG	pBO2860
CueR_dC5.rv			TTTTAAGCTTTCAACAGCAGCCGGAGAGATTTTC	
CueR-untagged_fw	*cueR*	gDNA	AAAAGCTAGCAACATCAGCGATGTAGCAAAAATTACC	pBO2862
CueR.rv			TTTTAAGCTTTCACCCTGCCCGATGATGAC	
CueR_ACYC.fw	*cueR*	gDNA	AAAAGATATCTAACAAAGCACAGGAGGCGTTGCG	pBO3687
CueR_ACYC.rv			AAAAGGATCCTCACCCTGCCCGATGATGA	
cueR_QC.fw	*cueR_C112S*	pBO2584	GCTAGCCCTGGCGATGACAGCGCCGACAGC	pBO4800
cueR_overlap_new.rv			CGCCAGGGCTAGCATTCGCCAGTGCCAGCAG	
cueR_QC_fwd	*cueR_C112S*	pBO3687	GCTAGCCCTGGCGATGACAGCGCCGACAGC	pBO4801
cueR_overlap_new.rv			CGCCAGGGCTAGCATTCGCCAGTGCCAGCAG	

### *In vivo* degradation experiments

To analyze the stability of different CueR variants, cells containing inducible expression plasmids encoding for corresponding CueR proteins were grown overnight in M9 minimal medium containing corresponding antibiotics for selection at 30°C. Fifteen milliliters of M9 minimal medium supplemented with corresponding antibiotics were inoculated with the overnight culture to an optical density (A_580_) of 0.05. Cells were grown to an A_580_ of 0.5 and protein expression was induced by adding 15 ng/ml anhydrotetracycline (AHT) for 20 min. Translation was blocked by addition of 200 μg/ml Cm. As an exception, translation of the strain lacking all three proteases (Δ*clpXP*, Δ*lon*, Δ*hslUV*) and its parental strain *E. coli* Wt MG1655 was blocked by addition of 300 μg/ml spectinomycin (Sp) since the triple knockout strain is resistant to Cm. Samples were taken at different time points, frozen into liquid nitrogen and subjected to SDS-PAGE, Western transfer, and immunodetection as described below.

To analyze the stability of Strep_CueR under defined copper concentrations the same *in vivo* degradation experiments were performed as described above with minor modifications: To avoid copper contamination all steps were performed in plastic ware and all M9 minimal medium components except trace elements were previously incubated overnight with 50 g/l Chelex 100 resin (Bio-Rad) to remove trace metals. Before usage trace metals (without copper component) were added to the medium, mixed and sterile-filtered. Cells were grown to an A_580_ of 0.5, defined copper concentrations (CuSO_4_) were supplemented for 1 h and the *in vivo* degradation experiments were performed as described above.

For analyses of Strep_CueR stability over the entire growth curve cells were grown in LB medium + Amp at 37°C to different growth phases and *in vivo* degradation experiments were performed in every growth phase as described above. To analyze Strep_CueR stability over the whole growth curve under different copper concentrations, defined copper concentrations (CuSO_4_) were added to the main cultures at the time of inoculation or a copper pulse was given to the main culture after the second *in vivo* degradation experiment had been started (~2.5 h after inoculation and 60 min before the third degradation experiment was started).

### Preparation of protein extracts and immunodetection

Cell pellets were resuspended in TE buffer depending on their optical density (10 mM Tris/HCl, pH 8; 1 mM EDTA; 50 μl TE buffer per A_580_ of 1.0) and mixed with protein sample buffer (final concentrations of 2% SDS (w/v), 0.1% (w/v) bromophenol blue, 10% (v/v) glycerol, 1% (v/v) β-mercaptoethanol, 50 mM Tris/HCl, pH 6.8). Samples were incubated for 5 min at 95°C, centrifuged (1 min, 16,000 × g) and subjected to SDS-PAGE and Western transfer using standard protocols (Sambrook and Russell, 2001). Strep-tagged fusion proteins were detected using a Strep-tag-HRP conjugate (IBA GmbH). Endogenous CueR and untagged CueR were detected using a polyclonal anti CueR antibody (Yamamoto and Ishihama, 2005) and a goat-anti-rabbit IgG (H+L) HRP conjugate (BioRad) as second antibody. Protein signals were visualized using Luminata Forte Western HRP substrate (Millipore) and the Chemi Imager Ready (Alpha Innotec). Half-lives of proteins were calculated by pixel counting with AlphaEaseFC software (version 4.0.0, Alpha Innotec).

### Protein purification

Strep_CueR (pBO2584), His_6__CspD (pBO1115), or Lon_His_6_ (pET21b-Lon) were transformed in *E. coli* Δ*lon*, BL21 or CH1019, respectively. Cells were grown to an A_580_ of 0.5 at 37°C in LB (Strep_CueR) or 2YT (His_6__CspD and Lon_His_6_) medium and gene expression was induced by addition of 150 ng/ml AHT (Strep_CueR) or 1 mM IPTG (isopropyl-β-D-thiogalactopyranoside) (His_6__CspD and Lon_His_6_). Cells were harvested after 3 h of overexpression at 30°C, resuspended in lysis buffer containing 20 mM Tris/HCl, pH 7.5, 200 mM NaCl, 1 mM DTT, 0.35 mg/ml lysozyme, 0.2 mg/ml DNase, and 0.2 mg/ml RNase and were disrupted via French Press. Strep- or His-tagged proteins were purified using streptactin sepharose (IBA GmbH) or Ni-NTA agarose (Qiagen), respectively. Purification of His-tagged proteins was performed as described previously (Langklotz and Narberhaus, 2011). Strep_CueR purification was performed using standard protocols of the purification kit (IBA GmbH). Protein concentrations were determined via Bradford assay (Bradford, 1976).

### *In vitro* degradation experiments

Fifteen micromolars of Strep_CueR or His_6__CspD and 600 nM Lon_His_6_ were incubated for 2 min at 37°C in the degradation buffer described in Bissonnette et al. (2010). *In vitro* degradation was initialized by addition of 20 mM ATP. Degradation experiments without addition of ATP were performed as controls. Results were visualized by SDS-PAGE and Coomassie staining or Western transfer following standard protocols (Sambrook and Russell, 2001).

### *In vivo* CueR activity assays

Cultures with inducible expression plasmids encoding different CueR variants were grown in plastic ware in copper-free M9 minimal medium treated with 50 g/l Chelex 100 resin (Bio-Rad) to remove trace metals. Before use trace metals (without copper component) and 30 ng/ml AHT were added to the medium, mixed and sterile-filtered. Cells were grown to an A_580_ of 0.5 and defined copper concentrations (CuSO_4_) were adjusted in the cultures. After 1 h, 1 ml of the culture was harvested for β-galactosidase activity assay. The assay was performed as described previously (Miller, 1972).

## Results and discussion

### CueR is a target of ATP-dependent proteolysis in *E. coli*

Transcriptional regulators differentially control genes in order to adapt the proteome to the ambient conditions. Both, level and activity of transcription regulators can be tuned to the cellular need. For instance, the basal level of the copper efflux regulator CueR always present in the cell is elevated at increasing copper concentrations (Yamamoto and Ishihama, 2005). The activity of transcriptional regulators is often controlled by modification or oligomerization. In case of CueR, only the Cu^+^-bound dimer (holo-CueR) is capable of activating expression of the copper tolerance genes *copA* and *cueO* (Outten et al., 2000). Just as important as activation of transcriptional regulators is their inactivation since the cell would waste valuable resources for expression of pathways not needed under the given condition. Moreover, uncontrolled overexpression of membrane proteins like CopA might compromise membrane integrity. Since CueR covalently binds Cu^+^ with high affinity in the zeptomolar range, it is unlikely that the transcription factor is inactivated by simple dissociation of copper from its metal-binding pocket (Changela et al., 2003). We postulate that *E. coli* might shut down the copper-stress response by proteolysis of the metal-loaded transcription factor.

**Figure 1:**
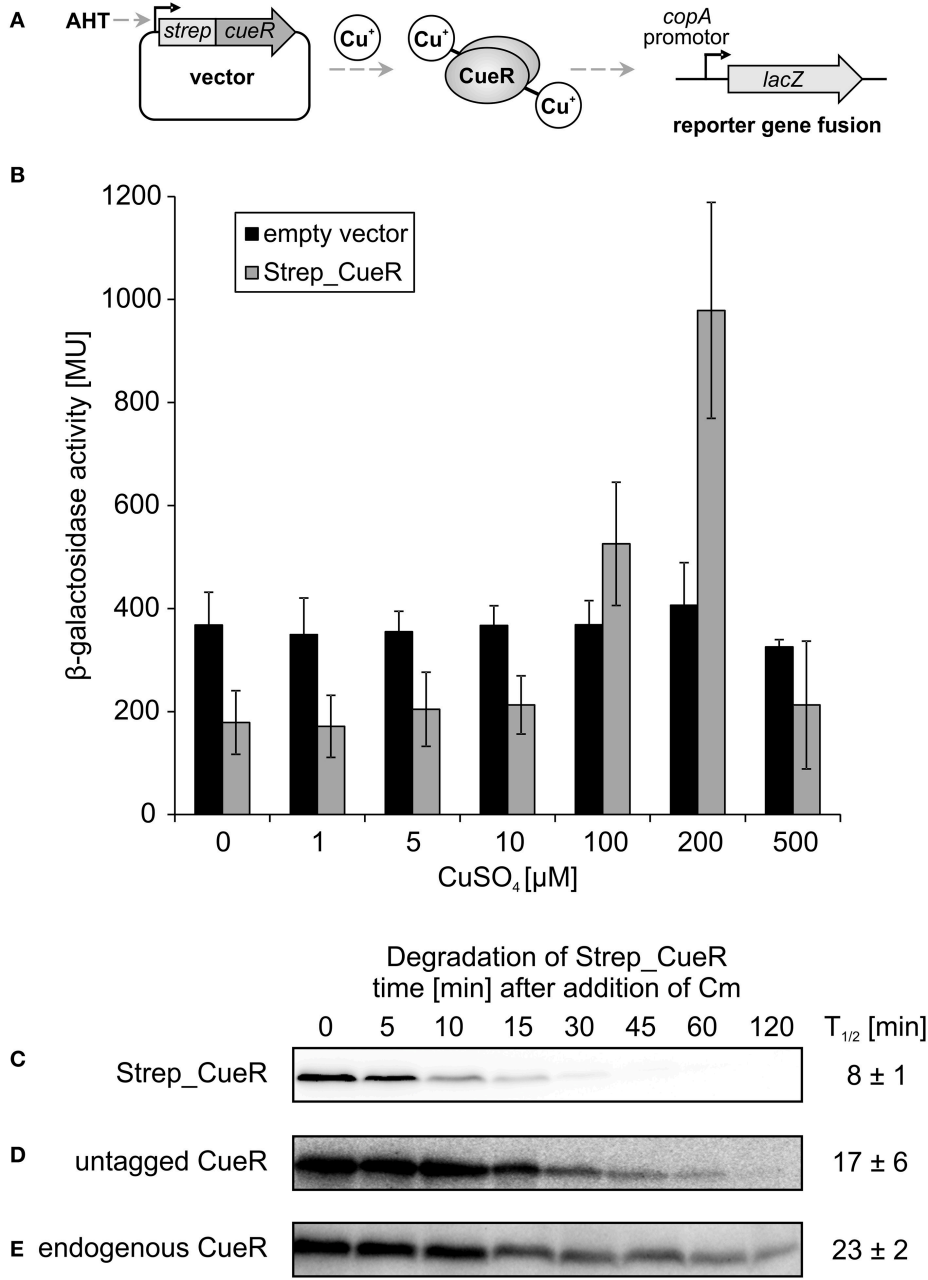
**Activity and stability of CueR in ***E. coli*****. Schematic presentation of the *in vivo* CueR activity assay **(A)**. *E. coli* Δ*cueR*, Φ(*copA-lacZ*) cells were transformed with the empty vector pASK-IBA5(+) or the inducible plasmid encoding Strep_CueR and grown to exponential growth phase (M9 minimal medium; with the addition of 30 ng/ml AHT; 30°C). Cells were stressed with increasing CuSO_4_ concentrations for 1 h and β-galactosidase activity was measured in Miller Units (MU). Standard deviations were calculated from at least two independent experiments **(B)**. Plasmid-encoded Strep_CueR was expressed for 20 min in exponential growth phase (M9 minimal medium; 30°C) in *E. coli* (MC4100). Translation was blocked by addition of Cm. Samples were taken at indicated time points, subjected to SDS-PAGE, Western transfer, and immunodetection. Half-lives (*T*_1/2_) and standard deviations were calculated from 10 independent experiments **(C)**. *In vivo* degradation experiments with plasmid-encoded untagged CueR were performed as described above. Half-lives (*T*_1/2_) and standard deviations were calculated from five independent experiments **(D)**. Stability of endogenous CueR was determined in *E. coli* MC4100 as described above. Half-lives (*T*_1/2_) and standard deviations were calculated from two independent experiments **(E)**.

To be able to address whether CueR is a protease substrate in *E. coli*, we expressed it as N-terminally Strep-tagged variant (Strep_CueR) that facilitates immunodetection of the protein. First, we used an activity assay previously described by Outten et al. to ascertain that the tagged protein is functionally active as transcription factor. The original assay is based on a Δ*cueR* strain encoding the CueR-dependent *copA* promoter fused to *lacZ* on the chromosome, and a plasmid encoding constitutively expressed *cueR* (Outten et al., 2000). To establish the assay we constitutively expressed untagged CueR and an inactive CueR variant (CueR_C112S) not able to bind Cu^+^ ions (Chen et al., 2003; Stoyanov and Brown, 2003). As expected, β-galactosidase activity increased with increasing copper concentration in the presence of CueR (Figure S1). The CueR_C112S variant was unable to activate *copA* expression and produced copper-independent background activity like the empty vector control strain (Figure S1). The assay worked equally well with Strep_CueR produced from an AHT-inducible plasmid (Figure 1A). Copper-controlled *copA* expression showed that the N-terminal Strep-tag did not interfere with transcriptional activation (Figure 1B). The stability of Strep_CueR was analyzed in an *E. coli* wildtype strain (MC4100) during exponential growth in M9 minimal medium after translation was blocked by addition of chloramphenicol. The protein was rapidly degraded with a half-life of about 8 min (Figure 1C) indicating that Strep_CueR is a target of proteolysis in *E. coli*. As control we performed *in vivo* degradation experiments with Strep_CueR in a strain lacking *cueR*, which had no effect on stability (Figure S2). Furthermore, both plasmid-encoded untagged and endogenous CueR were prone to proteolysis, yet with higher half-lives compared to the Strep-tagged version (Figures 1D,E). A similar effect on the half-life of tagged proteins was observed for the related transcription factor ZntR (Pruteanu et al., 2007).

### Strep_CueR is degraded by Lon, ClpXP and ClpAP

**Figure 2:**
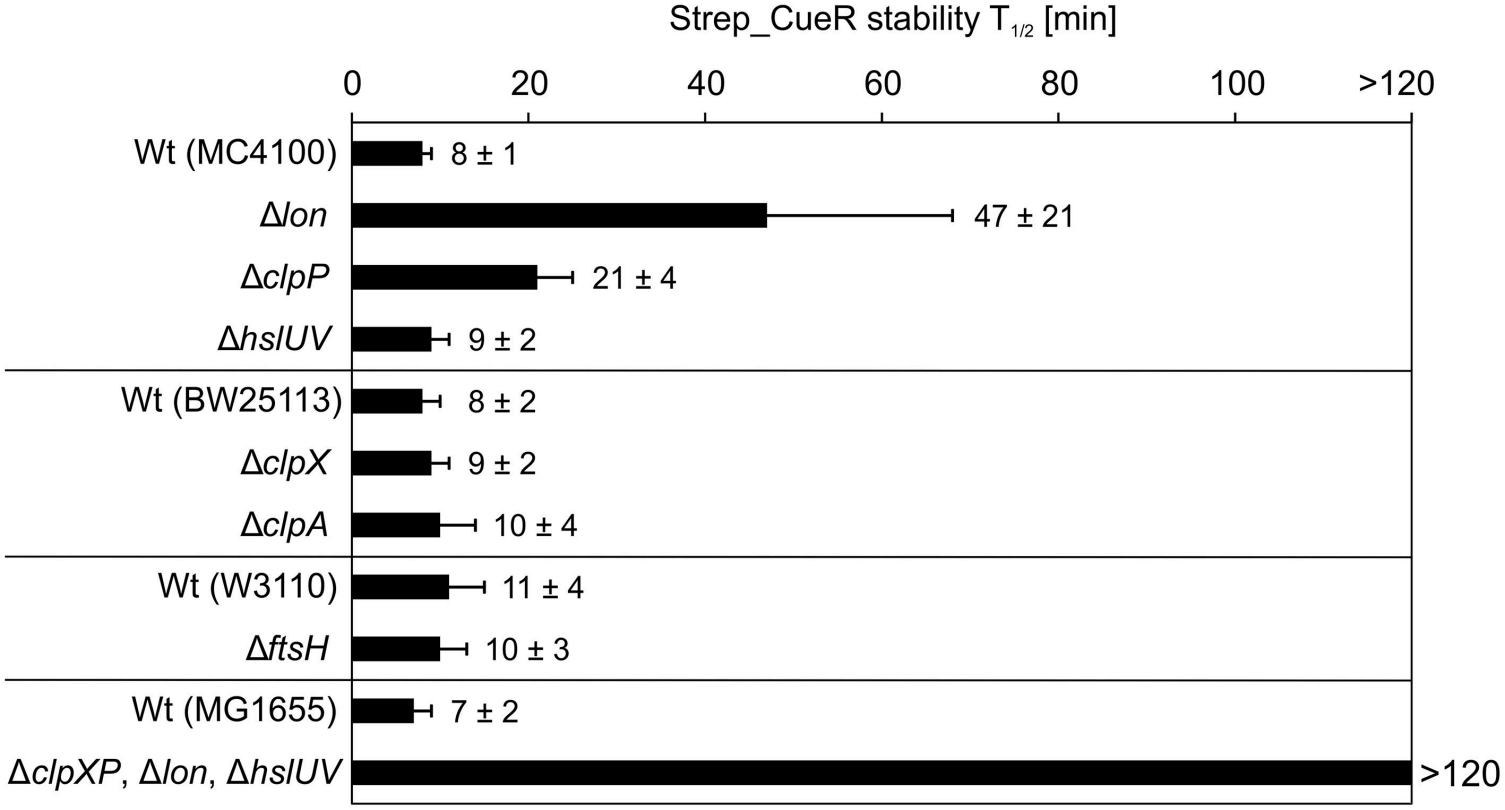
**Strep_CueR is degraded by Lon, ClpXP, and ClpAP protease**. Plasmid-encoded Strep_CueR was expressed for 20 min in exponential growth phase (M9 minimal medium; 30°C) in different protease-deficient *E. coli* strains and their corresponding wild-type (Wt) strains. Translation was blocked by addition of Cm or with spectinomycin for the strain lacking all three proteases (Δ*clpXP*, Δ*lon*, Δ*hslUV*) and its parental strain (MG1655) since the triple knockout strain is resistant to Cm. Samples were taken at indicated time points, subjected to SDS-PAGE, Western transfer, and immunodetection. Half-lives (*T*_1/2_) and standard deviations were calculated from at least two or three independent experiments.

To identify the protease responsible for Strep_CueR degradation, we monitored the stability of the protein in various protease-deficient *E. coli* strains and their corresponding parental strains. In all parental strains and in strains lacking the membrane-anchored FtsH (Δ*ftsH*) or the cytosolic HslUV (Δ*hslUV*) protease, the half-life of Strep_CueR was not altered. Hence, FtsH and HslUV are not involved in proteolysis of the transcription factor (Figure 2). In contrast, Strep_CueR was stabilized about sixfold in the Δ*lon* strain. Endogenous CueR also was equally stabilized with a half-life around 2 h in the *lon* mutant (Figure S3). In a strain lacking the proteolytic ClpP subunit of the ClpXP and ClpAP complexes Strep_CueR was stabilized about two to threefold. On the contrary, in strains lacking only one of the ATPases of the ClpP-containing proteases (either ClpX or ClpA) Strep_CueR was degraded wild-type-like suggesting that both ATPase subunits contribute to CueR proteolysis. As expected, Strep_CueR was completely stable in a strain void of all cytosolic AAA^+^ proteases (Figure 2). Substrate sharing by different AAA^+^ proteases has been described previously and contributes to robust post-translational regulation. For instance, the MerR family member ZntR is degraded by Lon and ClpXP but not by ClpAP (Pruteanu et al., 2007). It seems that regulated proteolysis of MerR-like regulators is a commonly used mechanism to control metal homeostasis in *E. coli*.

### Activity of CueR does not influence its stability

**Figure 3:**
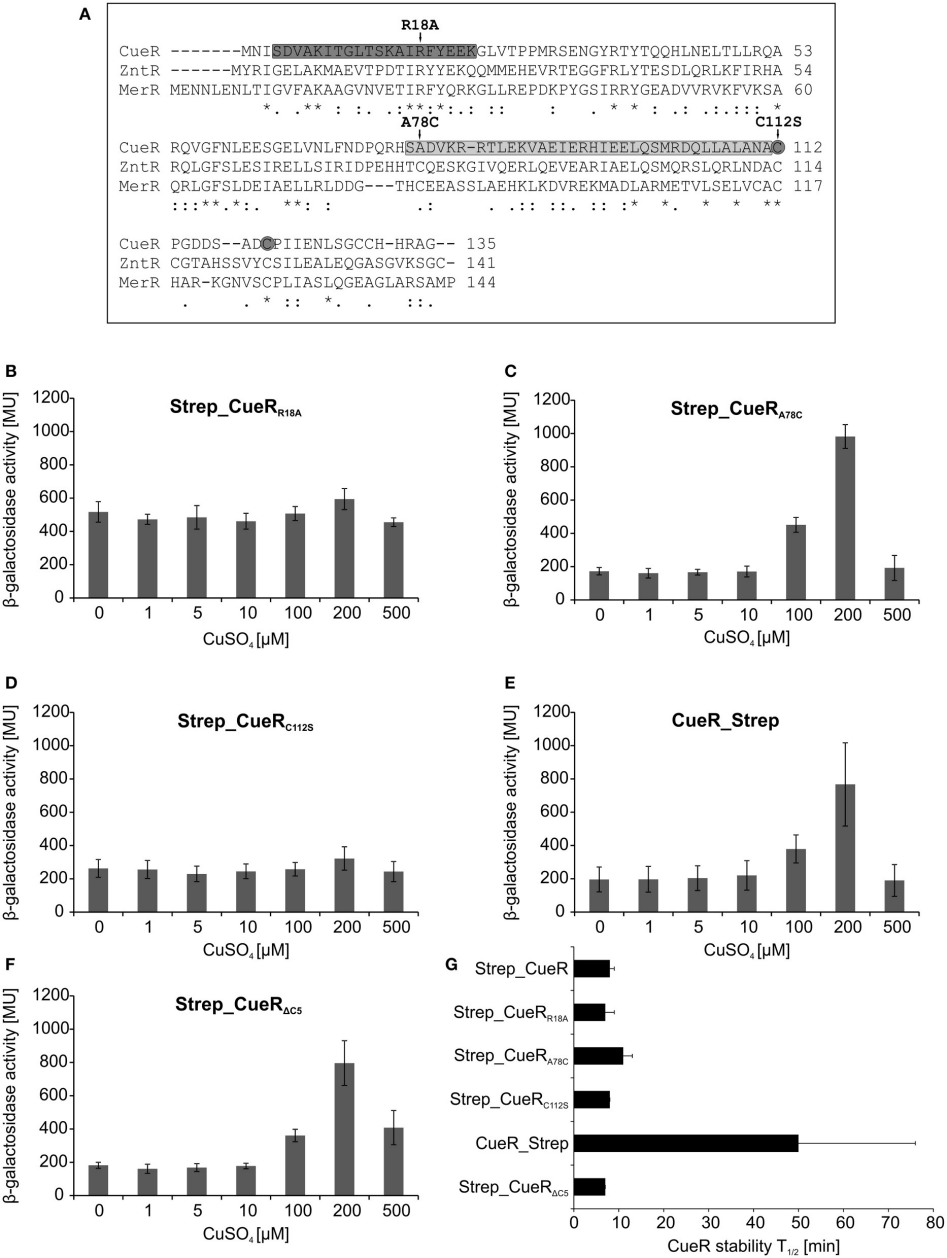
**Activity and stability of various CueR variants in ***E. coli*****. Comparison of the amino acid sequence of CueR, ZntR, and MerR. DNA-binding domain and dimerization domain of CueR are marked in dark gray and light gray, respectively. Amino acids, which were substituted in different variants used in this study, are highlighted with arrows and the two copper-binding cysteines of CueR are indicated with gray circles. (^*^ = identical amino acid; : = conserved substitution;. = semi conserved substitution; − = lacking amino acid). Alignment was performed by using the align tool of the uniprot database (http://www.uniprot.org/) **(A)**. *E. coli* Δ*cueR*, Φ(*copA-lacZ*) cells harboring inducible plasmids encoding Strep_CueR_R18A_ **(B)**, Strep_CueR_A78C_ **(C)**, Strep_CueR_C112S_ **(D)**, CueR_Strep **(E)**, or Strep_CueR_ΔC5_ **(F)** were grown in M9 minimal medium with 30 ng/ml AHT at 30°C to log phase. Cells were then treated with increasing CuSO_4_ concentrations for 1 h. β-galactosidase activity and standard deviations were calculated from at least two independent experiments **(B–F)**. Plasmid-encoded CueR variants were expressed for 20 min in exponential growth phase (M9 minimal medium; 30°C). Translation was blocked by addition of Cm. Samples were taken at indicated time points, subjected to SDS-PAGE, Western transfer, and immunodetection. Half-lives (*T*_1/2_) and standard deviations were calculated from at least three independent experiments. For comparison half-life of Strep_CueR is presented **(G)**.

Next, we analyzed whether already known recognition strategies of Lon or ClpP-containing AAA^+^ proteases apply to CueR. The mechanisms how AAA^+^ proteases recognize their substrates are highly diverse (Hoskins et al., 2002; Sauer et al., 2004; Baker and Sauer, 2006; Sauer and Baker, 2011). Lon predominantly recognizes proteins with exposed aromatic and hydrophobic residues as it is often the case in unfolded or unassembled proteins (Chung and Goldberg, 1981; Gur and Sauer, 2008). Terminal degrons recognized by Lon have also been identified (Ishii et al., 2000; Ishii and Amano, 2001; Shah and Wolf, 2006). Among them is the SsrA-tag, which is C-terminally added via the tmRNA system to polypeptides stalled during translation. However, SsrA-tagged proteins are predominantly recognized by ClpXP (Keiler et al., 1996; Flynn et al., 2001), a protease that is also known to utilize N-terminal degrons (Flynn et al., 2003). ClpAP recognizes several substrates via the so-called N-end rule pathway, in which the first N-terminal amino acid is critical for degradation (Erbse et al., 2006; Mogk et al., 2007; Dougan et al., 2010; Román-Hernández et al., 2011). Comparison of residues in the N or C terminus of CueR with known degrons of Lon, ClpXP and ClpAP did not reveal similarities to other protease substrates. The same was reported for the zinc-dependent transcriptional regulator ZntR, degraded by Lon and ClpXP in *E. coli* (Pruteanu et al., 2007) suggesting that yet unknown mechanisms of recognition may apply to these MerR-like proteins. For ZntR it was shown that mutation of the conserved arginine in the helix-turn-helix motif of the DNA-binding region results in faster degradation of the protein (Pruteanu et al., 2007). Therefore, we constructed a corresponding Strep_CueR_R18A_ variant (Figure 3A), which as expected (Philips et al., 2015) failed to induce *copA-lacZ* transcription since DNA binding is impaired (Figure 3B). Yet, degradation of the inactive CueR variant was not affected (Figure 3G and Figure S4).

Two additional variants of N-terminally Strep-tagged CueR with amino acid substitutions in functionally relevant regions of the protein were analyzed (Figure 3A). Strep_CueR_*A*78*C*_ is a variant with a substitution at the very beginning of the dimerization helix that differs from ZntR and MerR, which have a conserved cysteine at this position. Strep_CueR_C112S_ carries a substitution of a copper-binding cysteine in the metal-binding domain. Strep_CueR_A78C_ is able to activate *copA* expression in a copper-responsive manner (Figure 3C), while substitution of one of the two copper-binding cysteines (Strep_CueR_C112S_) inactivated CueR (Figure 3D). Regardless of whether they were active as transcription factor or not, both point-mutated variants were degraded like Strep_CueR (Figure 3G and Figure S4). Therefore, like for ZntR (Pruteanu et al., 2007), mutations in the dimerization and metal binding regions do not influence proteolysis.

Since some degrons are exposed at the termini of a substrate, we placed a Strep-tag at the C terminus to see whether it affects protein stability. Terminal tags have previously been shown to block proteolysis, for example of the Lon substrate SoxS (Griffith et al., 2004). Although activity of CueR_Strep was unaffected (Figure 3E), the protein was stabilized about six-fold (Figure 3G and Figure S4), suggesting a contribution of the C-terminal end to protease targeting. We also constructed a C-terminally truncated, active version of Strep_CueR lacking the last five C-terminal residues (Figure 3F). Strep_CueR_ΔC5_ was degraded like Strep_CueR (Figure 3G and Figure S4) excluding that the last five amino acids of the C terminus are critical for recognition.

**Figure 4:**
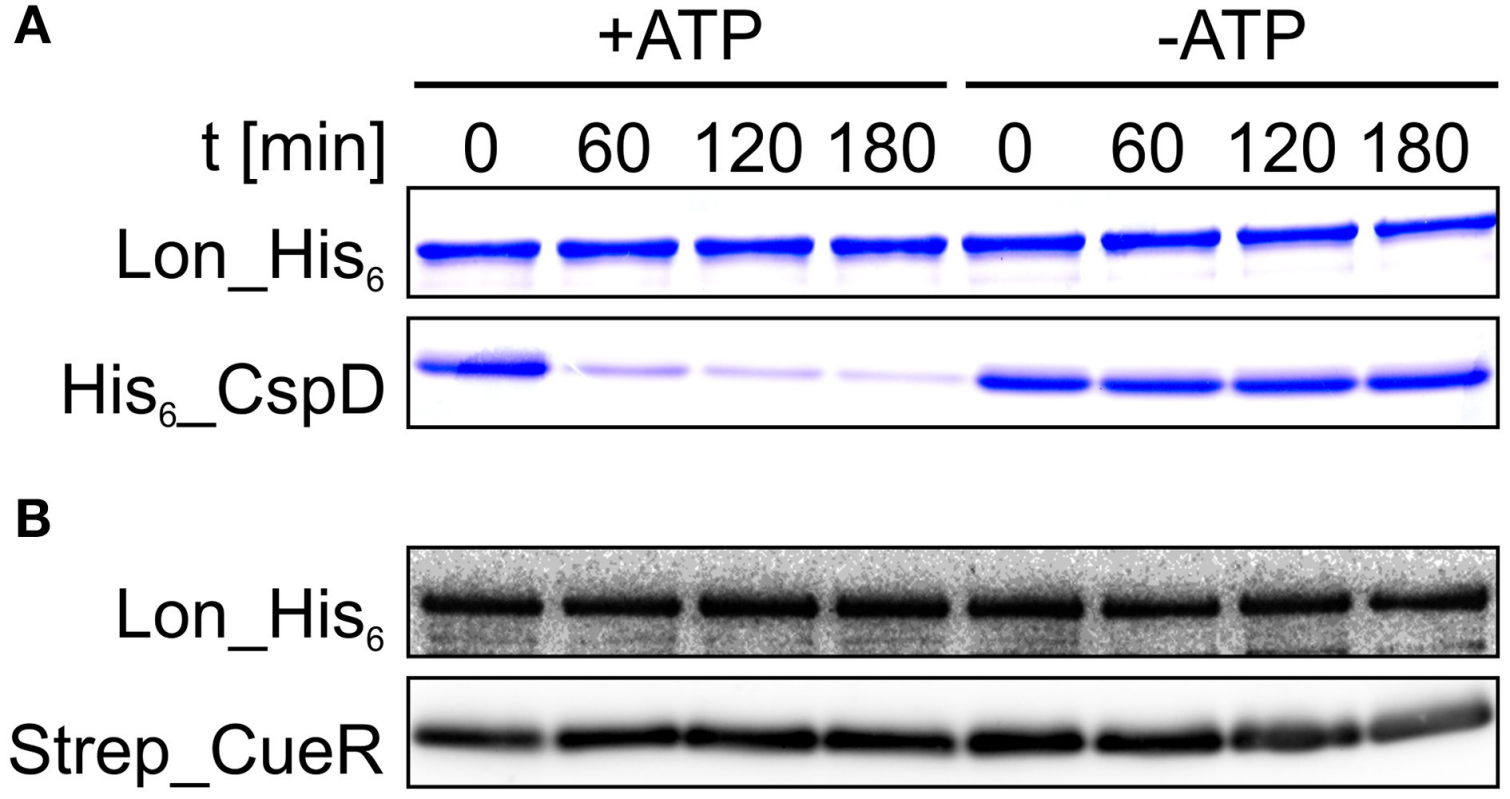
**Strep_CueR is not degraded by Lon ***in vitro*****. Lon_His_6_, His_6__CspD, and Strep_CueR were purified and used for *in vitro* degradation experiments **(A,B)**. Degradation experiments were initialized by addition of 20 mM ATP (+ATP). An approach without ATP addition (-ATP) served as control. Samples were taken at indicated time points, subjected to SDS-PAGE and Coomassie staining for His_6__CspD **(A)** or were subjected to Western transfer, and immunodetection for Strep_CueR **(B)**. Data are representative of five independent experiments.

Sometimes the recognition process is aided by adaptor proteins (Battesti and Gottesman, 2013). Given that Strep_CueR is primarily degraded by the Lon protease *in vivo* (Figure 2), we analyzed if it is degraded by Lon in a reconstituted *in vitro* system. For this purpose, Strep_CueR and Lon_His_6_ were purified and subjected to *in vitro* degradation experiments. The replication inhibitor His_6__CspD served as a control protein as it is known to be a direct substrate of Lon *in vitro* (Langklotz and Narberhaus, 2011) (Figure 4A). In contrast to His_6__CspD, Strep_CueR remained stable when incubated without (Figure S5) or with Lon_His_6_, both in the absence and presence of ATP (Figure 4B). This is in contrast to ZntR, which is degraded by Lon but not by ClpXP *in vitro* (Pruteanu et al., 2007). On the one hand it is possible that Lon needs to be allosterically activated for CueR degradation as it was shown for the replication initiator DnaA from *Caulobacter crescentus*. DnaA is degraded *in vivo* but remains stable in *in vitro* degradation experiments. When Lon is allosterically activated by the addition of unfolded proteins, DnaA is degraded *in vitro* (Jonas et al., 2013; Joshi and Chien, 2016). On the other hand a factor mediating Strep_CueR degradation might be missing in the purified system. Putative non-proteinaceous regulatory molecules might be guanosine pentaphosphate/tetraphosphate ((p)ppGpp) and inorganic poly phosphates (polyP), which are known to influence proteolysis of several AAA^+^ protease substrates (Kuroda et al., 2001, 2006; Kuroda, 2006; Schäkermann et al., 2013; Bittner et al., 2015). However, we can exclude an involvement of (p)ppGpp and polyP in CueR proteolysis since the protein was wild-type-like degraded in strains lacking these regulatory molecules (data not shown).

A putative adaptor protein lacking in the *in vitro* system might sense the cellular copper status. This is reminiscent of the adaptor protein YjbH that is able to coordinate zinc ions and is involved in ClpXP-dependent degradation of the transcriptional regulator Spx in *Bacillus subtilis* and *Staphylococcus aureus* (Garg et al., 2009; Engman et al., 2012). To date little is known about adaptors involved in Lon-dependent degradation. Recently, degradation of the master regulator of flagellar biosynthesis SwrA in *B. subtilis* was reported to be assisted by the swarming motility inhibitor A (SmiA), *in vivo* and *in vitro*. Hence, SmiA is the first described adaptor protein for Lon-dependent proteolysis (Mukherjee et al., 2015). Further, studies targeted at identifying the CueR degron and potential adaptor proteins might reveal similarities and differences in the recognition logics of ZntR and CueR.

### Is proteolysis of CueR regulated?

**Figure 5:**
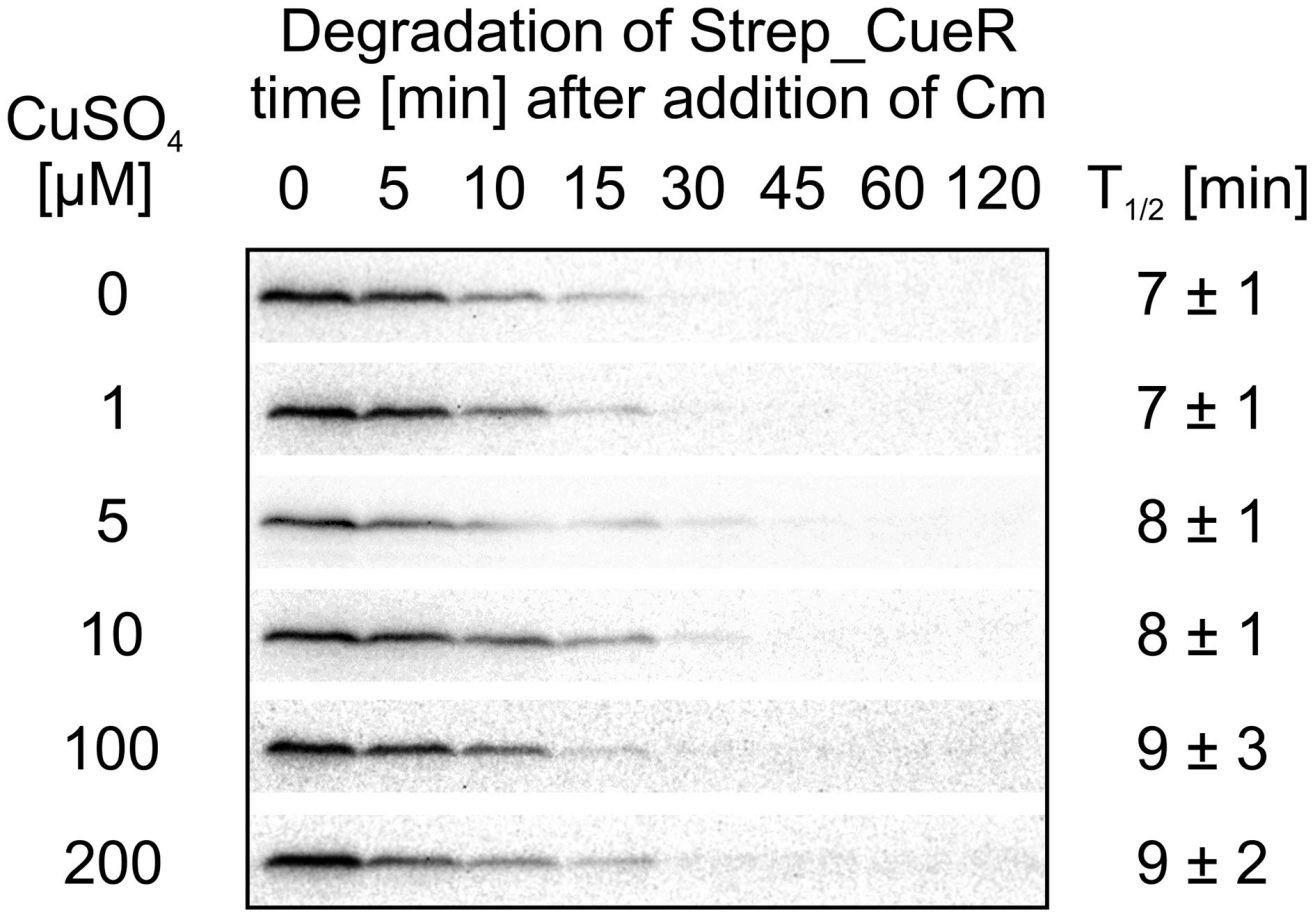
**Stability of Strep_CueR in response to increasing CuSO_**4**_ concentrations**. *E. coli* MC4100 (Wt) cells harboring a plasmid encoding for Strep_CueR were grown to exponential phase (M9 minimal medium at 30°C). Cultures were supplemented with varying CuSO_4_ concentrations for 1 h followed by *in vivo* degradation experiments. Translation was blocked by addition of Cm. Samples were taken at indicated time points, subjected to SDS-PAGE, Western transfer, and immunodetection. Half-lives (*T*_1/2_) and standard deviations were calculated from at least two independent experiments.

As proteolysis of some metalloregulators, like ZntR, Ctr1p, or Mac1 is metal-dependent (Ooi et al., 1996; Zhu et al., 1998; Liu et al., 2007; Pruteanu et al., 2007), we analyzed the effect of defined copper concentrations on the stability of Strep_CueR. *E. coli* cells harboring an AHT-inducible plasmid encoding Strep_CueR were grown to exponential growth phase under copper-limited conditions. Cells were then supplemented with various copper concentrations for 1 h followed by *in vivo* degradation experiments. The half-life of Strep_CueR remained similar at CuSO_4_ concentrations between 0 and 200 μM (Figure 5) indicating that the cellular copper level has little effect on CueR stability.

**Figure 6:**
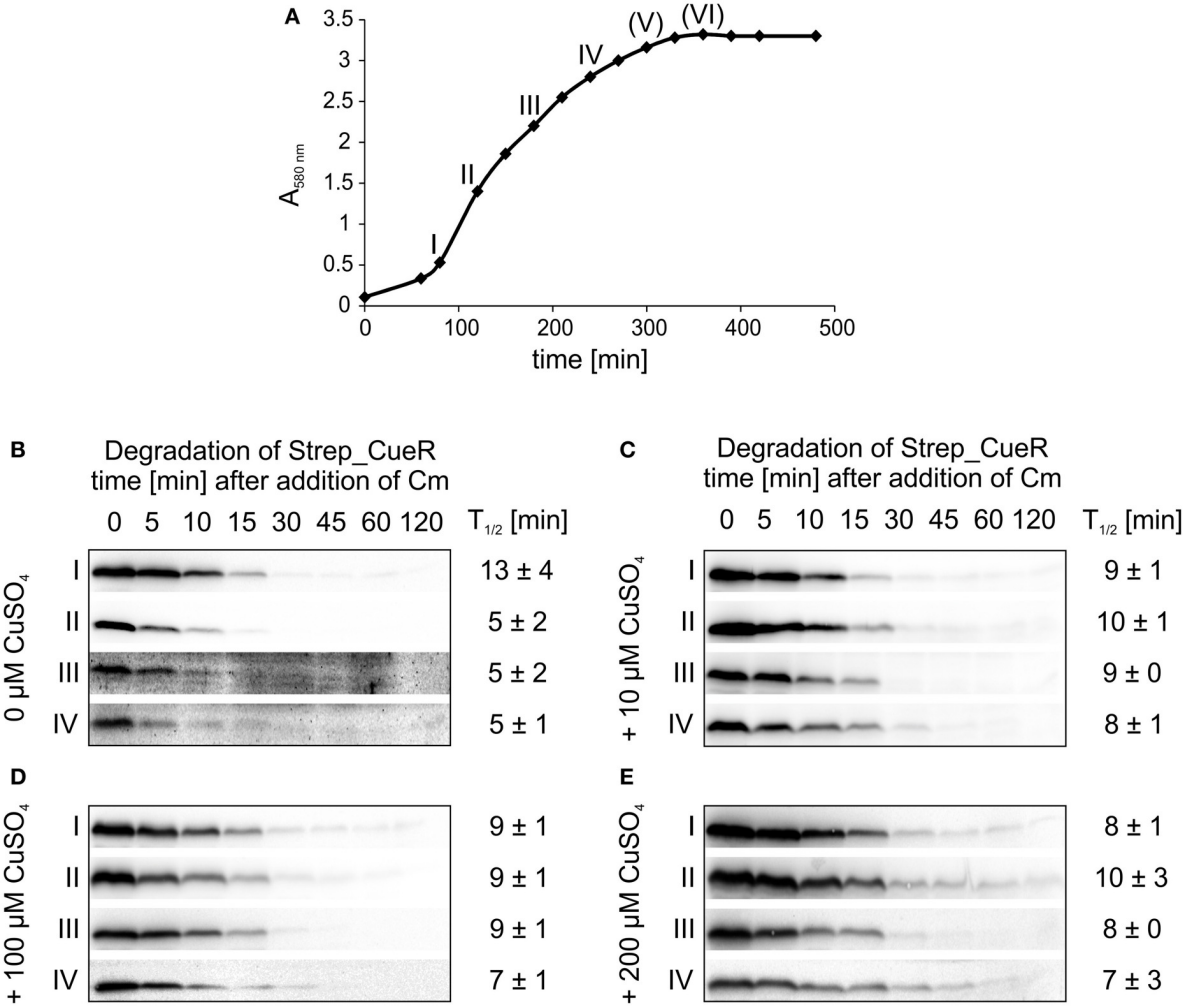
**Degradation of Strep_CueR in different growth phases under varying CuSO_**4**_ concentrations**. Stability of Strep_CueR was determined in LB medium at 37°C in different growth phases (I–VI) **(A)** in *E. coli* MC4100 (Wt) under varying CuSO_4_ concentrations **(B-E)**. Defined CuSO_4_ concentrations (0-200 μM) were added right from inoculation of the main culture. *In vivo* degradation experiments were performed after 20 min of Strep_CueR induction in every growth phase. Translation was blocked by addition of Cm. Samples were taken at indicated time points, subjected to SDS-PAGE, Western transfer, and immunodetection. Half-lives (*T*_1/2_) and standard deviations were calculated from at least two independent experiments. Strep_CueR was not detectable in *in vivo* degradation experiments (V) and (VI).

It recently turned out that degradation for several protease substrates is growth phase-dependent (Langklotz and Narberhaus, 2011; Westphal et al., 2012; Bittner et al., 2015). Therefore, we examined whether CueR stability depends on the growth status of *E. coli* and performed *in vivo* degradation experiments with Strep_CueR in different growth phases. All experiments described above were performed in M9 minimal medium at 30°C. To allow optimal growth, LB medium, and a temperature of 37°C were chosen for this experiment (Figure 6A). When no additional copper was added to the culture, degradation was accelerated about twofold from early exponential to exponential growth phase but remained the same in late exponential growth phase (Figure 6B). Strep_CueR was not detectable in later growth phases. Addition of external copper to the growth medium right from the beginning of the experiment led to constant half-lives in the range between 8 and 10 min (Figures 6C–E).

**Figure 7:**
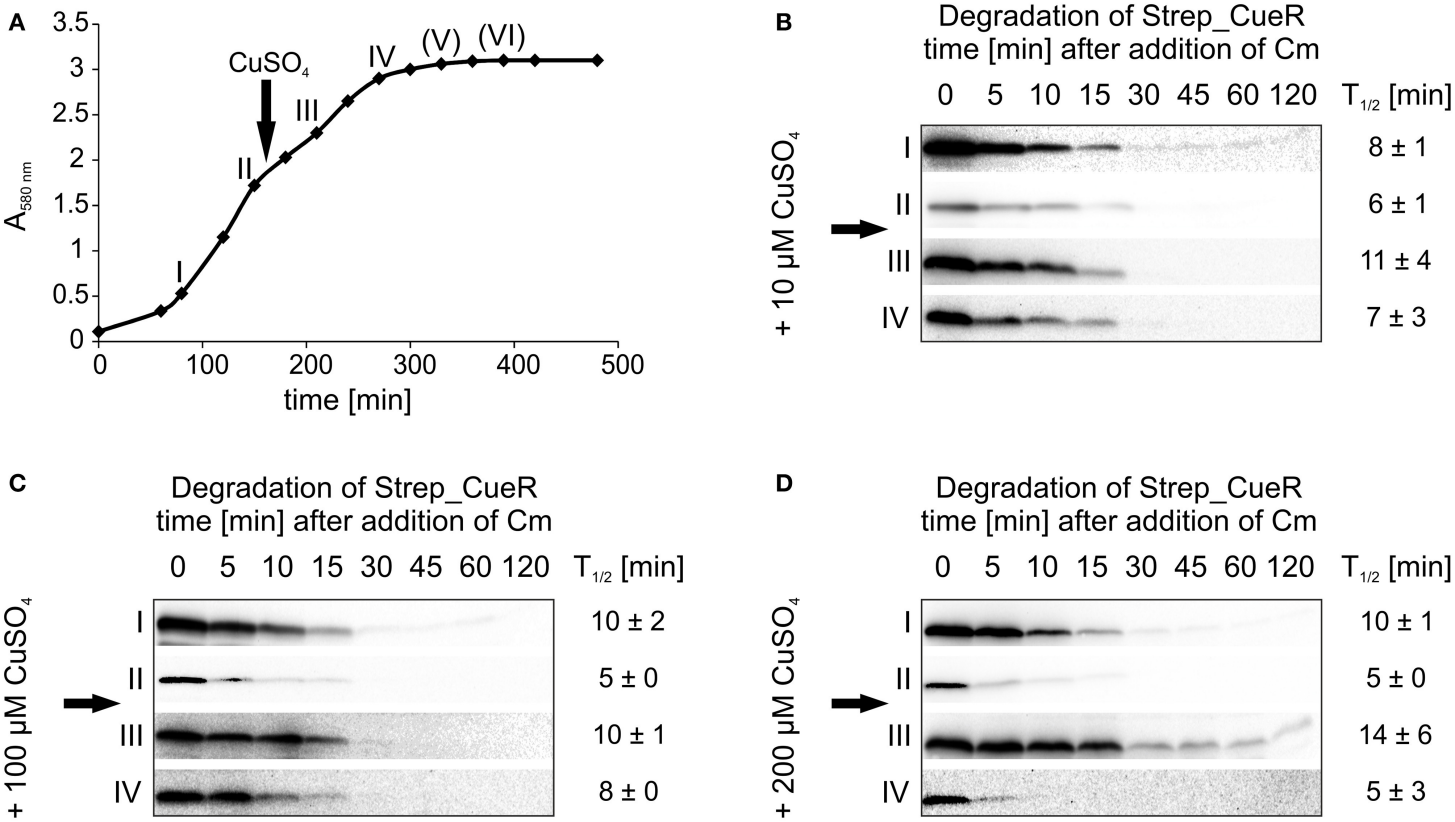
**Stability of Strep_CueR in different growth phases before and after a CuSO_**4**_ pulse**. Stability of Strep_CueR was determined in different growth phases in *E. coli* MC4100 (Wt) before and after a copper pulse (arrow) with different CuSO_4_ concentrations added to the main culture. Cells were grown to different growth phases (I-VI) in LB medium at 37°C **(A)**. The first two *in vivo* degradation experiments were performed without CuSO_4_ treatment (I and II) **(B–D)**. After 20 min of Strep_CueR induction in every growth phase, translation was blocked by addition of Cm. Samples were taken at indicated time points, subjected to SDS-PAGE, Western transfer, and immunodetection. Approx. 2.5 h after inoculation a CuSO_4_ pulse (10–200 μM CuSO_4_) was given to the main cultures and *in vivo* degradation experiments in further growth phases (III–VI) followed like described before. Half-lives (*T*_1/2_) and standard deviations were calculated from at least two independent experiments. Strep_CueR was not detectable in *in vivo* degradation experiments (V) and (VI).

To address whether sudden copper stress affects CueR degradation, we carried out *in vivo* degradation experiments over the entire growth curve with a copper pulse ~2.5 h after inoculation (Figure 7A). As shown above (Figure 6B), Strep_CueR showed a slightly accelerated degradation upon entry into exponential growth prior to copper treatment (Figures 7B–D; time points I and II). Immediately after a copper pulse of 10, 100, or 200 μM CuSO_4_, the stability of Strep_CueR increased about twofold before it returned to pre-shock values (Figures 7B–D, time points III and IV) indicating that the cells sensed and slightly reacted to altered copper concentrations. Again, the transcription factor was not detectable in late growth phases. Accelerated degradation of CueR in copper-starved fast-growing cells and transient stabilization of the protein after copper shock are consistent with the physiological demand for this copper export regulator. This is in good agreement with (i) ZntR, which is also only stabilized about two-fold after the addition of zinc (Pruteanu et al., 2007) and (ii) the estimation that newly synthesized CopA proteins reach sufficient efflux power about 2 min after addition of copper to clear excess copper from the cytosol (Tottey et al., 2007).

Overall, it seems that *E. coli* continuously degrades CueR with minor adjustments to the external copper status. We propose that this is due to the fast clearance of excess copper after CueR activation of the Cue system, which might not require a long-term stabilization of CueR. Secondly, proteolysis might preferentially erase the overrepresented copper-loaded form of CueR, thereby contributing to the maintenance of a copper-free CueR pool derived from new synthesis to allow measuring the acute copper level in the cell. Apo-CueR may exchange holo-CueR dimers bound to the corresponding promoters via the recently postulated mechanisms of direct substitution or assisted dissociation (Joshi et al., 2012; Chen et al., 2013). Both pathways are based on the formation of a very short-lived transition state (determined as Protein_2_-DNA ternary complex), in which two CueR dimers (e.g., apo- and holo-CueR) bind to the extended spacer sequence of the -35 and -10 regions of *copA* or *cueO* with one of their DNA-binding domains. Given the instability of this state, one CueR protein, e.g., holo-CueR, loses its grip on the dyad giving the other CueR dimer, apo-CueR, the chance to fully bind to the dyad with both of its DNA-binding domains (direct substitution) or both dimers fall off the DNA (assisted dissociation) (Joshi et al., 2012; Chen et al., 2013, 2015). The constitutive proteolysis of CueR described in this study might contribute to an accurate adjustment of the CueR pool always prepared to react to the current cellular copper level to efficiently maintain copper homeostasis.

## Author contributions

LB, SS, AK, and FN designed the study. LB and AK performed the experiments. LB and FN wrote the manuscript. All authors reviewed the results and approved the final version of the manuscript.

### Conflict of interest statement

The authors declare that the research was conducted in the absence of any commercial or financial relationships that could be construed as a potential conflict of interest.

## References

[B1] Baba T. Ara T. Hasegawa M. Takai Y. Okumura Y. Baba M.. (2006). Construction of *Escherichia coli* K-12 in-frame, single-gene knockout mutants: the Keio collection. Mol. Syst. Biol. 2:2006.0008. 10.1038/msb4100050 16738554 PMC1681482

[B2] Bachmann B. J. (1972). Pedigrees of some mutant strains of *Escherichia coli* K-12. Bacteriol. Rev. 36, 525–557. 4568763 10.1128/br.36.4.525-557.1972 PMC408331

[B3] Baker T. A. Sauer R. T. (2006). ATP-dependent proteases of bacteria: recognition logic and operating principles. Trends Biochem. Sci. 31, 647–653. 10.1016/j.tibs.2006.10.006 17074491 PMC2717004

[B4] Barembruch C. Hengge R. (2007). Cellular levels and activity of the flagellar sigma factor FliA of *Escherichia coli* are controlled by FlgM-modulated proteolysis. Mol. Microbiol. 65, 76–89. 10.1111/j.1365-2958.2007.05770.x 17537210

[B5] Battesti A. Gottesman S. (2013). Roles of adaptor proteins in regulation of bacterial proteolysis. Curr. Opin. Microbiol. 16, 140–147. 10.1016/j.mib.2013.01.002 23375660 PMC3646950

[B6] Becker G. Hengge-Aronis R. (2001). What makes an *Escherichia coli* promoter σ^*S*^ dependent? role of the -13/-14 nucleotide promoter positions and region 2.5 of σ^*S*^. Mol. Microbiol. 39, 1153–1165. 10.1111/j.1365-2958.2001.02313.x 11251833

[B7] Bissonnette S. A. Rivera-Rivera I. Sauer R. T. Baker T. A. (2010). The IbpA and IbpB small heat-shock proteins are substrates of the AAA^+^ Lon protease. Mol. Microbiol. 75, 1539–1549. 10.1111/j.1365-2958.2010.07070.x 20158612 PMC3934651

[B8] Bittner L. M. Arends J. Narberhaus F. (2016). Mini review: ATP-dependent proteases in bacteria. Biopolymers 105, 505-517. 10.1002/bip.22831 26971705

[B9] Bittner L. M. Westphal K. Narberhaus F. (2015). Conditional proteolysis of the membrane protein YfgM by the FtsH protease depends on a novel N-terminal degron. J. Biol. Chem. 290, 19367–19378. 10.1074/jbc.m115.648550 26092727 PMC4521054

[B10] Bradford M. M. (1976). A rapid and sensitive method for the quantitation of microgram quantities of protein utilizing the principle of protein-dye binding. Anal. Biochem. 72, 248–254. 10.1016/0003-2697(76)90527-3 942051

[B11] Brocklehurst K. R. Hobman J. L. Lawley B. Blank L. Marshall S. J. Brown N. L.. (1999). ZntR is a Zn(II)-responsive MerR-like transcriptional regulator of *zntA* in *Escherichia coli*. Mol. Microbiol. 31, 893–902. 10.1046/j.1365-2958.1999.01229.x 10048032

[B12] Brown N. L. Stoyanov J. V. Kidd S. P. Hobman J. L. (2003). The MerR family of transcriptional regulators. FEMS Microbiol. Rev. 27, 145–163. 10.1016/S0168-6445(03)00051-2 12829265

[B13] Changela A. Chen K. Xue Y. Holschen J. Outten C. E. O'Halloran T. V.. (2003). Molecular basis of metal-ion selectivity and zeptomolar sensitivity by CueR. Science 301, 1383–1387. 10.1126/science.1085950 12958362

[B14] Chen K. Yuldasheva S. Penner-Hahn J. E. O'Halloran T. V. (2003). An atypical linear Cu(I)-S2 center constitutes the high-affinity metal-sensing site in the CueR metalloregulatory protein. J. Am. Chem. Soc. 125, 12088–12089. 10.1021/ja036070y 14518983

[B15] Chen P. Keller A. M. Joshi C. P. Martell D. J. Andoy N. M. Benítez J. J.. (2013). Single-molecule dynamics and mechanisms of metalloregulators and metallochaperones. Biochemistry. 52, 7170–7183. 10.1021/bi400597v 24053279 PMC3830456

[B16] Chen T. Y. Santiago A. G. Jung W. Krzeminski L. Yang F. Martell D. J.. (2015). Concentration- and chromosome-organization-dependent regulator unbinding from DNA for transcription regulation in living cells. Nat. Commun. 6, 7445. 10.1038/ncomms8445 26145755 PMC4507017

[B17] Chivers P. T. (2007). A galvanizing story-protein stability and zinc homeostasis. J. Bacteriol. 189, 2953–2954. 10.1128/jb.00173-07 17307845 PMC1855864

[B18] Chung C. H. Goldberg A. L. (1981). The product of the *lon* (*capR*) gene in *Escherichia coli* is the ATP-dependent protease, protease La. Proc. Natl. Acad. Sci. U.S.A. 78, 4931–4935. 10.1073/pnas.78.8.4931 6458037 PMC320299

[B19] Dougan D. A. Truscott K. N. Zeth K. (2010). The bacterial N-end rule pathway: expect the unexpected. Mol. Microbiol. 76, 545–558. 10.1111/j.1365-2958.2010.07120.x 20374493

[B20] Engman J. Rogstam A. Frees D. Ingmer H. von Wachenfeldt C. (2012). The YjbH adaptor protein enhances proteolysis of the transcriptional regulator Spx in *Staphylococcus aureus*. J. Bacteriol. 194, 1186–1194. 10.1128/jb.06414-11 22194450 PMC3294810

[B21] Erbse A. Schmidt R. Bornemann T. Schneider-Mergener J. Mogk A. Zahn R.. (2006). ClpS is an essential component of the N-end rule pathway in *Escherichia coli*. Nature 439, 753–756. 10.1038/nature04412 16467841

[B22] Flynn J. M. Levchenko I. Seidel M. Wickner S. H. Sauer R. T. Baker T. A. (2001). Overlapping recognition determinants within the ssrA degradation tag allow modulation of proteolysis. Proc. Natl. Acad. Sci. U.S.A. 98, 10584–10589. 10.1073/pnas.191375298 11535833 PMC58509

[B23] Flynn J. M. Neher S. B. Kim Y. I. Sauer R. T. Baker T. A. (2003). Proteomic discovery of cellular substrates of the ClpXP protease reveals five classes of ClpX-recognition signals. Mol. Cell 11, 671–683. 10.1016/S1097-2765(03)00060-1 12667450

[B24] Garg S. K. Kommineni S. Henslee L. Zhang Y. Zuber P. (2009). The YjbH protein of *Bacillus subtilis* enhances ClpXP-catalyzed proteolysis of Spx. J. Bacteriol. 191, 1268–1277. 10.1128/jb.01289-08 19074380 PMC2632004

[B25] Grass G. Rensing C. (2001). CueO is a multi-copper oxidase that confers copper tolerance in *Escherichia coli*. Biochem. Biophys. Res. Commun. 286, 902–908. 10.1006/bbrc.2001.5474 11527384

[B26] Grass G. Rensing C. Solioz M. (2011). Metallic copper as an antimicrobial surface. Appl. Environ. Microbiol. 77, 1541–1547. 10.1128/aem.02766-10 21193661 PMC3067274

[B27] Griffith K. L. Shah I. M. Wolf R. E. Jr. (2004). Proteolytic degradation of *Escherichia coli* transcription activators SoxS and MarA as the mechanism for reversing the induction of the superoxide (SoxRS) and multiple antibiotic resistance (Mar) regulons. Mol. Microbiol. 51, 1801–1816. 10.1046/j.1365-2958.2003.03952.x 15009903

[B28] Gur E. Biran D. Ron E. Z. (2011). Regulated proteolysis in Gram-negative bacteria-how and when? Nat. Rev. Microbiol. 9, 839–848. 10.1038/nrmicro2669 22020261

[B29] Gur E. Ottofueling R. Dougan D. A. (2013). Machines of destruction - AAA+ proteases and the adaptors that control them. Subcell. Biochem. 66, 3–33. 10.1007/978-94-007-5940-4_1 23479435

[B30] Gur E. Sauer R. T. (2008). Recognition of misfolded proteins by Lon, a AAA^+^ protease. Genes Dev. 22, 2267–2277. 10.1101/gad.1670908 18708584 PMC2518814

[B31] Hoskins J. R. Yanagihara K. Mizuuchi K. Wickner S. (2002). ClpAP and ClpXP degrade proteins with tags located in the interior of the primary sequence. Proc. Natl. Acad. Sci. U.S.A. 99, 11037–11042. 10.1073/pnas.172378899 12177439 PMC123206

[B32] Ishii Y. Amano F. (2001). Regulation of SulA cleavage by Lon protease by the C-terminal amino acid of SulA, histidine. Biochem. J. 358, 473–480. 10.1042/bj3580473 11513747 PMC1222081

[B33] Ishii Y. Sonezaki S. Iwasaki Y. Miyata Y. Akita K. Kato Y.. (2000). Regulatory role of C-terminal residues of SulA in its degradation by Lon protease in *Escherichia coli*. J. Biochem. 127, 837–844. 10.1093/oxfordjournals.jbchem.a022677 10788793

[B34] Jonas K. Liu J. Chien P. Laub M. T. (2013). Proteotoxic stress induces a cell-cycle arrest by stimulating Lon to degrade the replication initiator DnaA. Cell 154, 623–636. 10.1016/j.cell.2013.06.034 23911325 PMC3749246

[B35] Joshi C. P. Panda D. Martell D. J. Andoy N. M. Chen T. Y. Gaballa A.. (2012). Direct substitution and assisted dissociation pathways for turning off transcription by a MerR-family metalloregulator. Proc. Natl. Acad. Sci. U.S.A. 109, 15121–15126. 10.1073/pnas.1208508109 22949686 PMC3458356

[B36] Joshi K. K. Chien P. (2016). Regulated Proteolysis in Bacteria: *Caulobacter*. Annu. Rev. Genet. 50, 423–445. 10.1146/annurev-genet-120215-035235 27893963 PMC5510660

[B37] Kanemori M. Yanagi H. Yura T. (1999). The ATP-dependent HslVU/CplQY protease participates in turnover of cell division inhibitor SulA in *Escherichia coli*. J. Bacteriol. 181, 3674–3680. 10368140 10.1128/jb.181.12.3674-3680.1999 PMC93843

[B38] Keiler K. C. Waller P. R. Sauer R. T. (1996). Role of a peptide tagging system in degradation of proteins synthesized from damaged messenger RNA. Science 271, 990–993. 10.1126/science.271.5251.990 8584937

[B39] Kuroda A. (2006). A polyphosphate-Lon protease complex in the adaptation of *Escherichia coli* to amino acid starvation. Biosci. Biotechnol. Biochem. 70, 325–331. 10.1271/bbb.70.325 16495646

[B40] Kuroda A. Nomura K. Ohtomo R. Kato J. Ikeda T. Takiguchi N.. (2001). Role of inorganic polyphosphate in promoting ribosomal protein degradation by the Lon protease in *E. coli*. Science 293, 705–708. 10.1126/science.1061315 11474114

[B41] Kuroda A. Nomura K. Takiguchi N. Kato J. Ohtake H. (2006). Inorganic polyphosphate stimulates Lon-mediated proteolysis of nucleoid proteins in *Escherichia coli*. Cell. Mol. Biol. 52, 23–29. 17543195

[B42] Langklotz S. Narberhaus F. (2011). The *Escherichia coli* replication inhibitor CspD is subject to growth-regulated degradation by the Lon protease. Mol. Microbiol. 80, 1313–1325. 10.1111/j.1365-2958.2011.07646.x 21435040

[B43] Liu J. Sitaram A. Burd C. G. (2007). Regulation of copper-dependent endocytosis and vacuolar degradation of the yeast copper transporter, Ctr1p, by the Rsp5 ubiquitin ligase. Traffic 8, 1375–1384. 10.1111/j.1600-0854.2007.00616.x 17645432

[B44] Lu Z. H. Dameron C. T. Solioz M. (2003). The *Enterococcus hirae* paradigm of copper homeostasis: copper chaperone turnover, interactions, and transactions. Biometals 16, 137–143. 10.1023/A:1020709307589 12572673

[B45] Lu Z. H. Solioz M. (2001). Copper-induced proteolysis of the CopZ copper chaperone of *Enterococcus hirae*. J. Biol. Chem. 276, 47822–47827. 10.1074/jbc.M106218200 11585824

[B46] Miller J. H. (1972). Experiments in Molecular Genetics. Harbor, NY: Cold Spring Harbor Laboratory Press.

[B47] Mogk A. Schmidt R. Bukau B. (2007). The N-end rule pathway for regulated proteolysis: prokaryotic and eukaryotic strategies. Trends Cell Biol. 17, 165–172. 10.1016/j.tcb.2007.02.001 17306546

[B48] Mukherjee S. Bree A. C. Liu J. Patrick J. E. Chien P. Kearns D. B. (2015). Adaptor-mediated Lon proteolysis restricts *Bacillus subtilis* hyperflagellation. Proc. Natl. Acad. Sci. U.S A. 112, 250–255. 10.1073/pnas.1417419112 25538299 PMC4291670

[B49] Ooi C. E. Rabinovich E. Dancis A. Bonifacino J. S. Klausner R. D. (1996). Copper-dependent degradation of the *Saccharomyces cerevisiae* plasma membrane copper transporter Ctr1p in the apparent absence of endocytosis. EMBO J. 15, 3515–3523. 8670854 PMC451948

[B50] Outten C. E. Outten F. W. O'Halloran T. V. (1999). DNA distortion mechanism for transcriptional activation by ZntR, a Zn(II)-responsive MerR homologue in *Escherichia coli*. J. Biol. Chem. 274, 37517–37524. 10.1074/jbc.274.53.37517 10608803

[B51] Outten F. W. Huffman D. L. Hale J. A. O'Halloran T. V. (2001). The independent *cue* and *cus* systems confer copper tolerance during aerobic and anaerobic growth in *Escherichia coli*. J. Biol. Chem. 276, 30670–30677. 10.1074/jbc.m104122200 11399769

[B52] Outten F. W. Outten C. E. Hale J. O'Halloran T. V. (2000). Transcriptional activation of an *Escherichia coli* copper efflux regulon by the chromosomal MerR homologue, *cueR*. J. Biol. Chem. 275, 31024–31029. 10.1074/jbc.M006508200 10915804

[B53] Petersen C. Møller L. B. (2000). Control of copper homeostasis in *Escherichia coli* by a P-type ATPase, CopA, and a MerR-like transcriptional activator, CopR. Gene 261, 289–298. 10.1016/S0378-1119(00)00509-6 11167016

[B54] Philips S. J. Canalizo-Hernandez M. Yildirim I. Schatz G. C. Mondragón A. O'Halloran T. V. (2015). Allosteric transcriptional regulation via changes in the overall topology of the core promoter. Science 349, 877–881. 10.1126/science.aaa9809 26293965 PMC4617686

[B55] Pruteanu M. Baker T. A. (2009). Proteolysis in the SOS response and metal homeostasis in *Escherichia coli*. Res. Microbiol. 160, 677–683. 10.1016/j.resmic.2009.08.012 19747971 PMC2783568

[B56] Pruteanu M. Neher S. B. Baker T. A. (2007). Ligand-controlled proteolysis of the *Escherichia coli* transcriptional regulator ZntR. J. Bacteriol. 189, 3017–3025. 10.1128/jb.01531-06 17220226 PMC1855835

[B57] Rademacher C. Masepohl B. (2012). Copper-responsive gene regulation in bacteria. Microbiology 158, 2451–2464. 10.1099/mic.0.058487-0 22918892

[B58] Rensing C. Fan B. Sharma R. Mitra B. Rosen B. P. (2000). CopA: An *Escherichia coli* Cu(I)-translocating P-type ATPase. Proc. Natl. Acad. Sci. U.S.A. 97, 652–656. 10.1073/pnas.97.2.652 10639134 PMC15385

[B59] Rensing C. Grass G. (2003). *Escherichia coli* mechanisms of copper homeostasis in a changing environment. FEMS Microbiol. Rev. 27, 197–213. 10.1016/S0168-6445(03)00049-4 12829268

[B60] Román-Hernández G. Hou J. Y. Grant R. A. Sauer R. T. Baker T. A. (2011). The ClpS adaptor mediates staged delivery of N-end rule substrates to the AAA+ ClpAP protease. Mol. Cell 43, 217–228. 10.1016/j.molcel.2011.06.009 21777811 PMC3168947

[B61] Sambrook J. Russell D. W. (2001). Molecular Cloning: A Laboratory Manual, 3rd Edn. Harbor, NY: Cold Spring Harbor Laboratory Press.

[B62] Sauer R. T. Baker T. A. (2011). AAA^+^ proteases: ATP-fueled machines of protein destruction. Annu. Rev. Biochem. 80, 587–612. 10.1146/annurev-biochem-060408-172623 21469952

[B63] Sauer R. T. Bolon D. N. Burton B. M. Burton R. E. Flynn J. M. Grant R. A.. (2004). Sculpting the proteome with AAA+ proteases and disassembly machines. Cell 119, 9–18. 10.1016/j.cell.2004.09.020 15454077 PMC2717008

[B64] Schäkermann M. Langklotz S. Narberhaus F. (2013). FtsH-mediated coordination of lipopolysaccharide biosynthesis in *Escherichia coli* correlates with the growth rate and the alarmone (p)ppGpp. J. Bacteriol. 195, 1912–1919. 10.1128/jb.02134-12 23417489 PMC3624583

[B65] Schmidt R. Zahn R. Bukau B. Mogk A. (2009). ClpS is the recognition component for *Escherichia coli* substrates of the N-end rule degradation pathway. Mol. Microbiol. 72, 506–517. 10.1111/j.1365-2958.2009.06666.x 19317833

[B66] Shah I. M. Wolf R. E. Jr. (2006). Sequence requirements for Lon-dependent degradation of the *Escherichia coli* transcription activator SoxS: identification of the SoxS residues critical to proteolysis and specific inhibition of *in vitro* degradation by a peptide comprised of the N-terminal 21 amino acid residues. J. Mol. Biol. 357, 718–731. 10.1016/j.jmb.2005.12.088 16460757

[B67] Solioz M. (2002). Role of proteolysis in copper homoeostasis. Biochem. Soc. Trans. 30, 688–691. 10.1042/bst0300688 12196165

[B68] Solioz M. Stoyanov J. V. (2003). Copper homeostasis in *Enterococcus hirae*. FEMS Microbiol. Rev. 27, 183–195. 10.1016/S0168-6445(03)00053-6 12829267

[B69] Stoyanov J. V. Brown N. L. (2003). The *Escherichia coli* copper-responsive *copA* promoter is activated by gold. J. Biol. Chem. 278, 1407–1410. 10.1074/jbc.C200580200 12446701

[B70] Stoyanov J. V. Hobman J. L. Brown N. L. (2001). CueR (YbbI) of *Escherichia coli* is a MerR family regulator controlling expression of the copper exporter CopA. Mol. Microbiol. 39, 502–511. 10.1046/j.1365-2958.2001.02264.x 11136469

[B71] Studier F. W. Rosenberg A. H. Dunn J. J. Dubendorff J. W. (1990). Use of T7 RNA-polymerase to direct expression of cloned genes. Methods Enzymol. 185, 60–89. 10.1016/0076-6879(90)85008-C 2199796

[B72] Tatsuta T. Tomoyasu T. Bukau B. Kitagawa M. Mori H. Karata K.. (1998). Heat shock regulation in the *ftsH* null mutant of *Escherichia coli*: dissection of stability and activity control mechanisms of σ^32^ *in vivo*. Mol. Microbiol. 30, 583–593. 10.1046/j.1365-2958.1998.01091.x 9822823

[B73] Tottey S. Harvie D. R. Robinson N. J. (2007). Understanding how cells allocate metals. Berlin; Heidelberg: Springer-Verlag. 10.1021/ar0300118 16231873

[B74] van der Oost J. de Boer A. P. de Gier J. W. Zumft W. G. Stouthamer A. H. van Spanning R. J. (1994). The heme-copper oxidase family consists of three distinct types of terminal oxidases and is related to nitric oxide reductase. FEMS Microbiol. Lett. 121, 1–9. 10.1111/j.1574-6968.1994.tb07067.x 8082820

[B75] Westphal K. Langklotz S. Thomanek N. Narberhaus F. (2012). A trapping approach reveals novel substrates and physiological functions of the essential protease FtsH in *Escherichia coli*. J. Biol. Chem. 287, 42962–42971. 10.1074/jbc.m112.388470 23091052 PMC3522291

[B76] Yamamoto K. Ishihama A. (2005). Transcriptional response of *Escherichia coli* to external copper. Mol. Microbiol. 56, 215–227. 10.1111/j.1365-2958.2005.04532.x 15773991

[B77] Zhu Z. Labbé S. Peña M. M. Thiele D. J. (1998). Copper differentially regulates the activity and degradation of yeast Mac1 transcription factor. J. Biol. Chem. 273, 1277–1280. 10.1074/jbc.273.3.1277 9430656

