# Supplementary material for: The Copper Efflux Regulator CueR Is Subject to ATP-Dependent Proteolysis in Escherichia coli
Source: Front Mol Biosci. 2017 Feb 28;4:9. doi: 10.3389/fmolb.2017.00009 (PMC5329002; doi:10.3389/fmolb.2017.00009)
Supplement: Supplementary file 1 [file DataSheet1.pdf]

Supporting information for:

The Copper Efflux Regulator CueR Is Subject to ATP-Dependent Proteolysis in *Escherichia coli*

Lisa-Marie Bittner[1], Alexander Kraus[1], Sina Schäkermann[1], and Franz Narberhaus[1]*

[1]Microbial Biology, Ruhr University Bochum, Bochum, Germany

*** Correspondence:**
Franz Narberhaus
franz.narberhaus@rub.de

Experimental procedures

CueR activity assays – *E. coli* Δ*cueR*, Φ(*copA-lacZ*) cells (Outten *et al.*, 2000) were transformed with plasmids encoding constitutively expressed CueR, CueR_C112S or the empty vector (pACYC184). Cultures were grown in plastic ware in copper-free M9 minimal medium treated with 50 g/liter Chelex 100 resin (Bio-Rad) to remove trace metals. Before usage trace metals (without copper component) were added to the medium, mixed and sterile-filtered. Cells were grown to an optical density (A_{580}) of 0.5 at 30 °C, defined copper concentrations ($CuSO_4$) were added to the cultures for 1 h and 1 ml culture was harvested for β-galactosidase activity assay. The assay was performed as described previously (Miller, 1972).

In vivo degradation experiments – To analyze the stability of Strep_CueR in the *cueR* mutant, corresponding cells containing an inducible expression plasmid encoding for Strep_CueR were grown overnight in M9 minimal medium containing corresponding antibiotics for selection at 30 °C. 15 ml M9 minimal medium supplemented with corresponding antibiotics were inoculated with the overnight culture to an optical density (A_{580}) of 0.05. Cells were grown to an A_{580} of 0.5 and protein expression was induced by adding 15 ng/ml anhydrotetracycline (AHT) for 20 min. Translation was blocked by addition of 200 µg/ml Cm. Samples were taken at different time points, frozen in liquid nitrogen and subjected to SDS-PAGE, Western transfer and immunodetection as described below. To analyze the stability of endogenous CueR in the *lon* mutant, *E. coli* Δ*lon* cells were grown in M9 minimal medium to an optical density (A_{580}) of 0.05 and *in vivo* degradation experiments were performed as described above.

Preparation of protein extracts and immunodetection – Cell pellets were resuspended in TE buffer depending on their optical density (10 mM Tris/HCl, pH 8; 1 mM EDTA; 50 µl TE buffer per A_{580} of 1.0) and mixed with protein sample buffer (final concentrations of 2 % SDS (w/v), 0.1 % (w/v) bromophenol blue, 10 % (v/v) glycerol, 1 % (v/v) β-mercaptoethanol, 50 mM Tris/HCl, pH 6.8). Samples were incubated for 5 min at 95 °C, centrifuged (1 min, 16,000 × g) and subjected to SDS-PAGE and Western transfer using standard protocols (Sambrook & Russell, 2001). Strep-tagged fusion proteins were detected using a Strep-tag-HRP conjugate (IBA GmbH). Protein signals were visualized using Luminata Forte Western HRP substrate (Millipore) and the Chemi Imager Ready (Alpha Innotec). Half-lives of proteins were calculated by pixel counting with AlphaEaseFC software (version 4.0.0, Alpha Innotec).

In vitro degradation experiments – 15 µM of Strep_CueR were incubated for 2 min at 37 °C in the degradation buffer described in (Bissonnette *et al.*, 2010). *In vitro* degradation was initialized by addition of 20 mM ATP. Degradation experiments without addition of ATP were performed as controls. Results were visualized by SDS-PAGE and Western transfer using standard protocols (Sambrook & Russell, 2001).

Figure S1

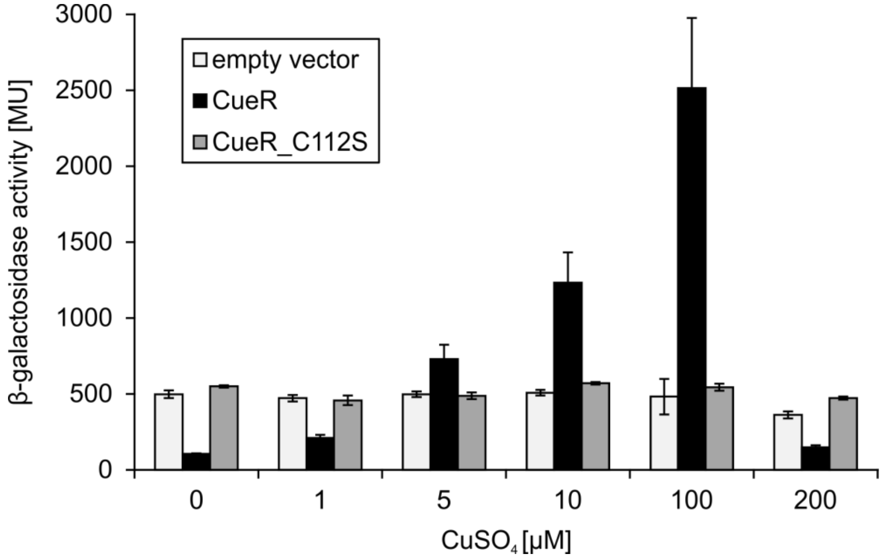

Figure S1. Effect of increasing $CuSO_4$ concentrations on CueR activity. *E. coli* Δ*cueR*, Φ(*copA-lacZ*) cells were transformed with plasmids encoding constitutively expressed CueR, CueR_C112S or the empty vector (pACYC184) and were grown to exponential growth phase in M9 minimal medium at 30 °C. Cells were stressed with increasing $CuSO_4$ concentrations for 1 h and β-galactosidase activity was measured in Miller Units (MU). Standard deviations were calculated from at least two independent experiments.

Figure S2

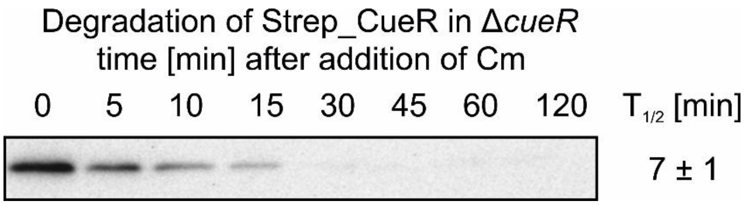

Figure S2. Plasmid-encoded Strep_CueR was expressed for 20 min in exponential growth phase (M9 minimal medium; 30 °C) in the *cueR* mutant. Translation was blocked by addition of Cm. Samples were taken at indicated time points, subjected to SDS-PAGE, Western transfer, and immunodetection. Half-lives ($T_{1/2}$) and standard deviations were calculated from three independent experiments.

Figure S3

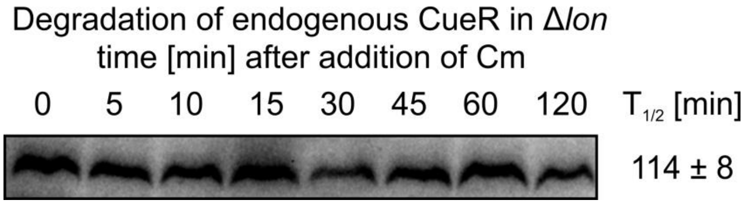

Figure S3. *E. coli* Δ*lon* cells were grown to exponential growth phase (M9 minimal medium; 30 °C). Translation was blocked by addition of Cm. Samples were taken at indicated time points, subjected to SDS-PAGE, Western transfer, and immunodetection. Half-lives of endogenous CueR ($T_{1/2}$) and standard deviations were calculated from two independent experiments.

Figure S4

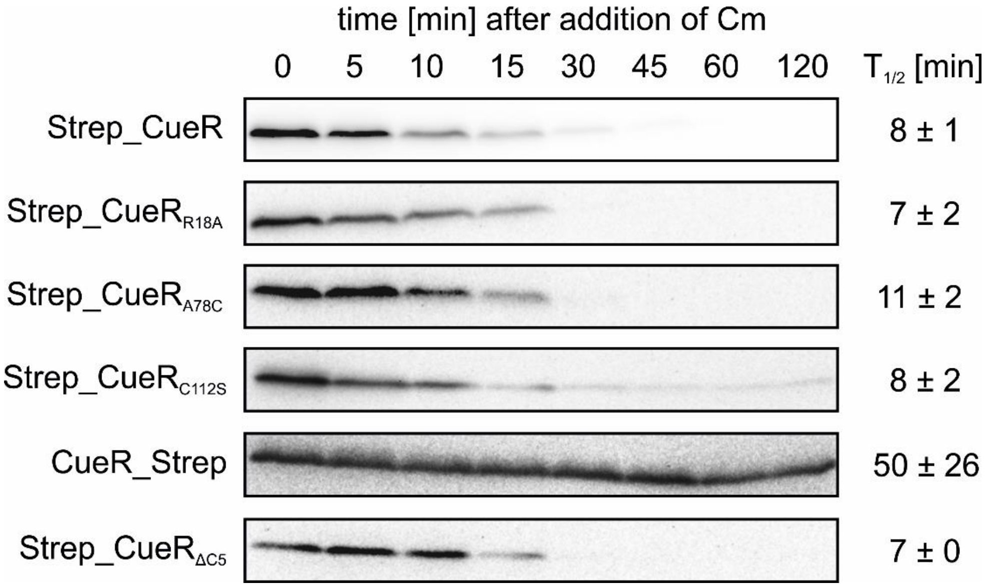

Figure S4. Stability of various CueR variants in *E. coli*.
Plasmid-encoded CueR variants were expressed for 20 min in exponential growth phase (M9 minimal medium; 30 °C). Translation was blocked by addition of Cm. Samples were taken at indicated time points, subjected to SDS-PAGE, Western transfer, and immunodetection. Half-lives ($T_{1/2}$) and standard deviations were calculated from at least three independent experiments. For comparison the Western blot for Strep_CueR was taken from Figure 1C.

Figure S5

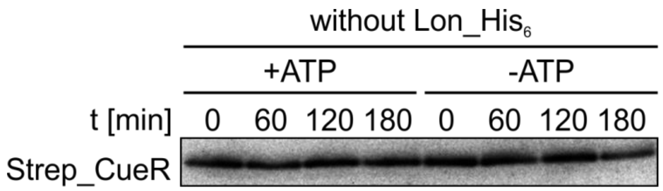

Figure S5. Strep_CueR is stable *in vitro*.
Strep_CueR was purified and used for a control *in vitro* degradation experiment without the presence of the Lon protease. The effect of ATP addition (+ATP) or the approach without ATP (-ATP) was analyzed. Samples were taken at indicated time points, subjected to SDS-PAGE, Western transfer, and immunodetection. Data are representative of two independent experiments.

References

Bissonnette, S. A., Rivera-Rivera I., Sauer R. T. & Baker T. A., (2010) The IbpA and IbpB small heat-shock proteins are substrates of the AAA^+ Lon protease. *Mol. Microbiol.* 75: 1539-1549. 10.1111/j.1365-2958.2010.07070.x.

Miller, J. H., (1972) Experiments in Molecular Genetics, Cold Spring Harbor Laboratory Press, Cold Spring Harbor, NY.

Outten, F. W., Outten C. E., Hale J. & O'Halloran T. V., (2000) Transcriptional activation of an *Escherichia coli* copper efflux regulon by the chromosomal MerR homologue, *cueR*. *J. Biol. Chem.* 275: 31024-31029. 10.1074/jbc.M006508200.

Sambrook, J. & Russell D. W., (2001) Molecular cloning: A Laboratory Manual, 3rd ed., Cold Spring Harbor Laboratory Press, Cold Spring Harbor, NY.
